# Impact of Age-3 Urine Screening on Diagnosis and Treatment Timing in Alport Syndrome

**DOI:** 10.1016/j.ekir.2025.09.022

**Published:** 2025-09-23

**Authors:** Hideaki Kitakado, Shingo Ishimori, Shuhei Aoyama, Yuka Kimura, Yuta Inoki, Chika Ueda, Yu Tanaka, Tomoko Horinouchi, Tomohiko Yamamura, Nana Sakakibara, China Nagano, Kandai Nozu

**Affiliations:** 1 Department of Pediatrics, Kobe University Graduate School of Medicine, Hyogo, Japan

**Keywords:** age-3 urine screening, Alport syndrome, early intervention, genetic test, hematuria

## Abstract

**Introduction:**

Alport syndrome is an inherited kidney disease that leads to end-stage kidney disease (ESKD) due to pathogenic variants in *COL4A3*/*4*/*5*, which encode type IV collagen. Promptly identifying patients with Alport syndrome and starting treatment with a renin–angiotensin system inhibitor (RAS-I) is important for delaying progression to ESKD. In Japan, public urine screening is available for all children aged 3 years.

**Methods:**

Patients genetically diagnosed with Alport syndrome at our department between August 2015 and May 2024, who were aged ≤ 18 years, were included in the study. We evaluated their clinical and genetic characteristics and identified the circumstances under which abnormal urine findings were first detected, with a focus on the role of urine screening at the age of 3 years (age-3 urine screening).

**Results:**

A total of 356 patients with Alport syndrome were eligible for this study. The most common setting for detecting urine abnormalities for the first time was during age-3 urine screening (*n* = 113, 31.7%). The inherited forms were as follows: X-linked (XL) female (43.3%), XL male (30.1%), autosomal dominant (AD) (19.5%), and autosomal recessive (AR) (6.2%) Alport syndrome. In addition, 60.2% of these patients already met the criteria for RAS-I treatment at the time of urine screening.

**Discussion:**

Our study showed that approximately 30% of patients with Alport syndrome had the opportunity to be diagnosed through age-3 urine screening, and among them, more than half were already eligible for RAS-I treatment. Urine screening may be an optimal method for contributing to a delay in the progression to ESKD in patients with Alport syndrome.

## Graphical abstract

**Figure:**
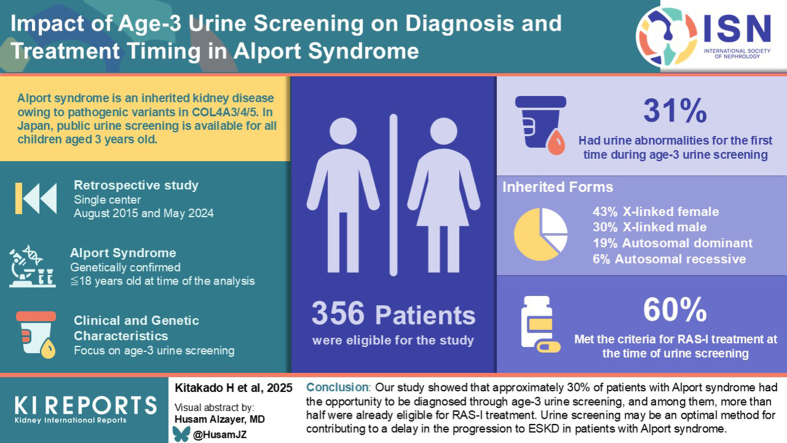

See Commentary on Page 4132

Alport syndrome is an inherited kidney disease leading to ESKD and is caused by the pathogenic variants of *COL4A3/4/5,* which encode type IV collagen. Alport syndrome has a prevalence of approximately 1 in 5000.^1^ Although patients with Alport syndrome have asymptomatic urinary abnormalities in early childhood, those with any types of inheritance modes present with a progressive decline in kidney function, although a different slope of decline is observed in each type.^1^, ^2^, ^3^, ^4^ Available data on the age of onset of hematuria in XL Alport syndrome (male or female), AD Alport syndrome, AR Alport syndrome, and digenic Alport syndrome are limited; however, most reports indicate that hematuria typically presents during early childhood. In contrast, more consistent evidence exists regarding the onset of proteinuria, with reported median ages of 6.5 years in AR Alport syndrome,^5^ 7 years in XL Alport syndrome (male or female),^6^ 10 years in digenic Alport syndrome,^7^ and 17 years in AD Alport syndrome,^8^ demonstrating a correlation between earlier onset and greater disease severity across subtypes. The primary complaints for detecting Alport syndrome are usually hematuria,^9^ a family history of Alport syndrome,^10^ and progressive hearing impairment.^11^ Regardless of the trigger for detection, because urinary abnormalities develop at a young age, the earlier these abnormalities are detected in their urine the earlier they are diagnosed.

Gross *et al.*^12^^,^^13^ showed that earlier intervention may improve kidney outcomes in pediatric patients with Alport syndrome who are aged > 2 years. Clinicians need to diagnose patients with Alport syndrome at the earliest possible age because earlier intervention can delay progression to chronic kidney disease and ESKD. In Japan, a urine screening program for all children aged 3 years was initiated in 1961 as part of a health checkup. In this screening, testing for proteinuria is mandatory. In contrast, the inclusion of hematuria and urinary β2-microglobulin measurements are determined by each municipality, resulting in a lack of standardization.^14^^,^^15^ Children with abnormal urinalysis findings are initially referred to their primary care provider or a nearby health care facility. If subsequent comprehensive evaluations, such as repeated urinalysis, laboratory blood tests, blood pressure measurements, and ultrasonographic examinations, continue to reveal abnormalities, these patients are then referred to specialized medical centers for further management.^14^ In other countries, a considerable proportion of patients with Alport syndrome present with kidney dysfunction or ESKD at the time of diagnosis.^16^, ^17^, ^18^, ^19^ In contrast, the age-3 urine screening system in Japan is thought to facilitate earlier detection and improve prognostic outcomes; however, its detailed implications remain unclear. Therefore, this study aimed to examine the impact of age-3 urine screening on diagnosis and treatment timing in children with Alport syndrome. We focused on patients with Alport syndrome in whom urinary abnormalities were first identified during age-3 urine screening. We investigated the proportion of cases and clinical characteristics of patients who were genetically diagnosed with Alport syndrome in our institute.

## Methods

### Patients

Patients diagnosed with genetically confirmed Alport syndrome at our department between August 2015 and May 2024, who were aged ≤ 18 years at the time of analysis, were included in the study. We aimed to collect cases of Alport syndrome under pediatric management because these were considered to provide easier access to information from early childhood. However, once patients transition to adult service, their information is often not transferred properly. Data regarding the patient’s background, how Alport syndrome was diagnosed, the genetic form, and clinical symptoms were extracted from the clinical information survey form completed at the time of genetic testing. In this study, proteinuria was defined as increased levels of protein in the urine, with a protein-to-creatinine ratio > 0.15 g/g of creatinine. In this study, the index event was defined as the initial detection of any urinary abnormality. An example of this definition is if gross hematuria was observed at an infantile stage and subsequent abnormal urinalysis findings were detected during nursery school screening at the age of 4 years, gross hematuria at an infantile stage was designated as the index event. Similarly, in patients in whom hematuria was identified during the age-3 urine screening, and hematuria and proteinuria were detected during the school screening, the former was adopted as the index event.

### Ethical Considerations

All procedures in this study were conducted according to the ethical standards of the Institutional Review Board of Kobe University School of Medicine (approval number: 301), the principles of the Declaration of Helsinki, and ethical guidelines of the Japanese Ministry of Health, Labour and Welfare. Informed consent was obtained from all patients and/or their parents.

### Genetic Analysis

We conducted a genetic analysis as previously reported.^20^, ^21^, ^22^ Briefly, genomic DNA was extracted from peripheral blood leukocytes obtained from patients using the QuickGene-Mini 80 System or QuickGene-Auto 12S (Kurabo Industries Ltd., Tokyo, Japan), according to the manufacturer’s instructions. Some patients had variants identified through direct sequencing of *COL4A3/4/5*. To perform next-generation sequencing, library preparation was conducted using the HaloPlex Target Enrichment System Kit (Agilent Technologies, Santa Clara, CA) according to the manufacturer’s instructions. *COL4A3/4/5* and other podocyte-related genes were sequenced using the MiSeq next-generation sequencing platform (Illumina, San Diego, CA). Sequenced data were aligned to the reference human genome (GRCh38/Hg19) and analyzed with SureCall 4.2.1.10. The patients were further analyzed in accordance with the American College of Medical Genetics guidelines,^23^^,^^24^ and judged whether the cases were pathogenic or likely pathogenic/uncertain significance. Initially, all patients were suspected of having Alport syndrome clinically and/or pathologically and were referred for a genetic test. The indications for a genetic test were at least one of the following: (i) a thin basement membrane, stratification, and/or reticular changes in the glomerular basement membrane observed on electron microscopy; (ii) abnormal alpha 5 staining patterns on the glomerular basement membrane; and (iii) a family history of Alport syndrome, chronic kidney disease, or suspected Alport syndrome clinically.

## Results

### Patients

**Figure 1:**
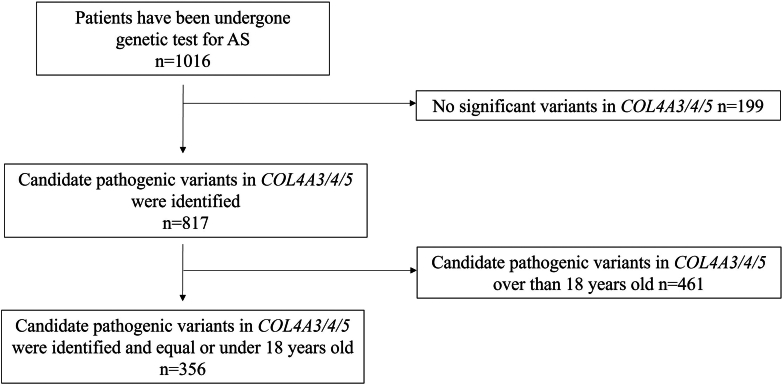
Flow chart of patients. Three hundred fifty-six cases aged < 18 years have candidate pathogenic variants. AS, Alport syndrome.

The number of patients who underwent a genetic test for Alport syndrome was 1016. Among 817 patients who had candidate pathogenic variants in *COL4A3/4/5*, we excluded 461 patients aged > 18 years at the timing of the genetic analysis. The remaining 356 patients were aged ≤ 18 years (Figure 1). The most common reason for referral to the hospital among these 356 patients was age-3 urine screening results (31.7%), followed by macroscopic hematuria (22.8%), and incidental detection of urinary abnormalities during the examination of other diseases (21.3%) (Table 1).

**Table 1:** Indication for referral to nephrology department

Events	Number, *n*	Ratio, %
Age-3 urine screening	113	31.7
Macroscopic hematuria	81	22.8
Incidental detection	76	21.3
Other urinalysis	73	20.5
Family history	9	2.5
Others	4	1.1
Total	356	100

### Clinical Characteristics

The clinical characteristics of all Alport syndrome cases are shown in Table 2. Among these, the cases detected through age-3 urine screening for the first time are shown in Table 3 and Supplementary Table S1. Among the cases of Alport syndrome detected through an age-3 urine screening, the number of patients with hematuria and proteinuria at this time who were referred to the hospital was 113 (100%) and 54 (47.8%), respectively. The genetic forms detected through age-3 urine screening were as follows: female XL Alport syndrome (43.4%), male XL Alport syndrome (30.1%), AD Alport syndrome (19.5%), AR Alport syndrome (6.2%), and digenic Alport syndrome (0.9%). The proportions of patients with hematuria and proteinuria at the time of age-3 urine screening were as follows: 100% and 48.4% in male XL Alport syndrome, 100% and 38.3% in female XL Alport syndrome, 100% and 85.6% in AR Alport syndrome, and 100% and 28.6% in AD Alport syndrome, respectively (Table 3).

**Table 2:** The background of all cases diagnosed with Alport syndrome by the age of 18 yrs

	All cases (*N* = 356)
Background	
Male sex, *n* (%)	184 (51.7)
Family history of	
End-stage kidney disease, *n* (%)	120 (33.7)
Alport syndrome, *n* (%)	39 (11.0)
Any nephritis, *n* (%)	29 (8.1)
Hematuria, *n* (%)	226 (63.5)
No Family history, *n* (%)	73 (20.5)
Complications	
Deafness, *n* (%)	26 (7.3)
Any ocular complications, *n* (%)	11 (3.1)
Histopathological findings	
Collagen IV chain α5 staining^a^, *n* (%)	113/151 (74.8)
Electron microscopy^b^, *n* (%)	175/184 (95.1)
At the timing of occasion referred to hospital	
Median age, yr	3 (1–3)
Hematuria, *n* (%)	356 (100)
Proteinuria, *n* (%)	154 (43.3)
At the timing of diagnosis genetically	
Median age, yr	7 (4–12)
Hematuria, *n* (%)	356 (100)
Proteinuria, *n* (%)	256 (71.9)
Urine protein-to-Cr ratio, (g/gCr)	0.32 (0.15–0.76)
Cr-eGFR < 90 (ml/min per 1.73 m^2^), *n* (%)	29 (8.1)
Genotype	
XL Alport syndrome/male, *n* (%)	143 (40.2%)
XL Alport syndrome/female, *n* (%)	127 (35.7%)
AD Alport syndrome, *n* (%)	58 (16.6%)
AR Alport syndrome, *n* (%)	23 (6.5%)
Digenic, *n* (%)	5 (1.4%)

AD, autosomal dominant; AR, autosomal recessive; Cr, creatinine; eGFR, estimated glomerular filtration rate; XL, X-linked.

a Complete or mosaic loss in collagen IV chain α5 staining.

b Lamination, thinning, and any other irregular thickness.

**Table 3:** The background of cases detected through age-3 urine screening

	age-3 urine screening (*n* = 113)
Background	
Male sex, *n* (%)	49 (43.4)
Family history of	
End-stage kidney disease, *n* (%)	34 (22.4)
Alport syndrome, *n* (%)	12 (10.6)
Any nephritis, *n* (%)	9 (8.0)
Hematuria, *n* (%)	71 (62.8)
No Family history, *n* (%)	24 (21.2)
Complications	
Deafness, *n* (%)	5 (4.4)
Any ocular complications, *n* (%)	2 (1.8)
Histopathological findings	
Collagen IV chain α5 staining^a^, *n* (%)	36/49 (74.5)
Electron microscopy^b^, *n* (%)	60/62 (96.8)
At the timing of occasion referred to hospital	
Median age, yr	3 (3–3)
Hematuria, *n* (%)	113 (100)
Proteinuria, *n* (%)	54 (47.8)
At the timing of diagnosis genetically	
Median age, yr	6 (4–12)
Hematuria, *n* (%)	113 (100)
Proteinuria, *n* (%)	78 (70.1)
Urine protein-to-Cr ratio, (g/gCr)	0.27 (0.13–0.57)
Cr-eGFR < 90 (ml/min per 1.73 m^2^), *n* (%)	8 (7.1)
Genotype	
XL Alport syndrome/male, *n* (%)	34 (30.1%)
XL Alport syndrome/female, *n* (%)	49 (43.4%)
AD Alport syndrome, *n* (%)	22 (19.5%)
AR Alport syndrome, *n* (%)	7 (6.2%)
Digenic, *n* (%)	1 (0.9%)

AD, autosomal dominant; AR: Autosomal recessive; Cr, creatinine, eGFR, estimated glomerular filtration rate; XL, X-linked.

a Complete or mosaic loss in collagen IV chain α5 staining.

b Lamination, thinning, and any other irregular thickness.

The number of patients with an indication for RAS-I therapy for Alport syndrome according to the Japanese Society of Pediatric Nephrology and Alport Classification Working Group^25^ was as follows: 34 (100%) in male XL Alport syndrome; 20 (38.3%) in female XL Alport syndrome; 7 (100%) in AR Alport syndrome; 6 (28.6%) in AD Alport syndrome, and 1 (100%) in digenic Alport syndrome. Sixty-eight of 113 patients (60.2%) who had Alport syndrome detected through age-3 urine screening met the indication of RAS-I therapy according to the guidelines (Table 4).

**Table 4:** Urinary findings and the distribution of therapy indication in each group divided according to genetic form

	XL Alport syndrome male (*n* = 34)	XL Alport syndrome female (*n* = 49)	AD Alport syndrome (*n* = 22)	AR Alport syndrome (*n* = 7)	Digenic (*n* = 1)	Total (*N* = 113)
At the time of age-3 urinalysis						
Hematuria, *n* (%)	34 (100)	49 (100)	22 (100)	7 (100)	1 (100)	113 (100)
Proteinuria, *n* (%)	18 (52.9)	20 (40.8)	6 (27.3)	6 (85.7)	0 (0)	45 (42.1)
Urine protein to-Cr ratio (g/gCr)	0.30 (0.15–1.0)	0.27 (0.13–0.41)	0.17 (0.12–0.32)	0.35 (0.24–1.99)	0.12 (-)	0.27 (0.13–0.53)
Cr-eGFR < 90 (ml/min per 1.73 m^2^), *n* (%)	4 (11.8)	3 (6.1)	1 (4.5)	0 (0)	0 (0)	8 (7.1)
Indications for starting RAS-I by JSPN/ACWG, *n* (%)	34 (100)	20 (40.8)	6 (27.3)	7 (100)	1 (100)	68 (60.2)
Indications for starting RAS-I by EARLY PRO-TECT Alport trial, *n* (%)	34 (100)	49 (100)	22 (100)	7 (100)	1 (100)	113 (100)

ACWG, Alport Syndrome Classification Working Group^18^; AD, autosomal dominant; AR, autosomal recessive; Cr, creatinine; EARLY PRO-TECT, The Early Prospective Therapy European Community Trial; eGFR, estimated glomerular filtration rate; JSPN, Japanese Society for Pediatric Nephrology^18^; RAS-I, renin-angiotensin system inhibitor; XL, X-linked.

## Discussion

In our study, we found that age-3 urine screening was the most common diagnostic setting for genetically diagnosed patients with Alport syndrome in Japan. Among the patients with Alport syndrome who were identified through age-3 urine screening, > 40% presented with proteinuria, and all of them had hematuria. Therefore, the implementation of urine screening can identify a considerable number of children with Alport syndrome who already show an indication for treatment. In addition, approximately 60% of patients who were detected through the age-3 urine screening met the indication for an RAS-I at the time of detection according to the guidelines. This finding indicates that a certain number of asymptomatic patients with Alport syndrome were already eligible for therapy at the age of 3 years. Consequently, the introduction of a urine testing system may allow for intervention in some Alport syndrome cases before the onset of proteinuria and kidney dysfunction. Among patients detected through age-3 urine screening, the proportion of patients with kidney dysfunction at the time of diagnosis was markedly lower than that previously reported.^7^^,^^8^^,^^20^^,^^26^^,^^27^

In male patients with XL Alport syndrome, the proportion of those with hematuria, proteinuria, kidney dysfunction, and ESKD at the point of diagnosis (median age: 13 years)^20^ was reported to be 100%, 95%, 77%, and 55%, respectively; and it was 100%, 67%, 36%, and 11%^28^ in female patients with XL Alport syndrome (median age: 24 years), respectively.^26^ The proportion of patients with AR Alport syndrome and hematuria, proteinuria, kidney dysfunction, and ESKD at the point of diagnosis (median age: 18.5 years) was reported to be 100%, 100%, 87%, and 43%, respectively^27^; and it was 100%, 72%, 33%, and 12%, respectively in those with AD Alport syndrome (median age: 53 years)^8^^,^^29^; and 100%, 86.7%, 81%, and 34.8%, respectively, in those with digenic Alport syndrome (median age: 34 years).^7^In our study, male patients with XL Alport syndrome and those with AR Alport syndrome (i.e., those lacking a wild-type allele) were more likely to meet treatment criteria at the time of diagnosis. This finding is consistent with previous reports that disease severity and earlier onset of proteinuria are associated with the absence of a normal *COL4A3/4/5* allele. The initiation of a RAS-I can prolong the timing at which patients reach ESKD.^20^^,^^30^^,^^31^ The Alport Classification Working Group have established indications of RAS-I therapy for patients with Alport syndrome as follows: male patients with XL Alport syndrome and AR Alport syndrome at the timing of diagnosis; and female patients with XL Alport syndrome and AD Alport syndrome at the timing of diagnosis with hematuria and proteinuria.^25^ The Japanese Society of Pediatric Nephrology follows this indication.^32^

Generally, only patients with Alport syndrome with a family history of this condition have the opportunity for urine tests at an early age in countries without any urine screening system. To achieve an early diagnosis in patients without a family history of Alport syndrome, promptly detecting any preceding urinary abnormalities is crucial, and conducting genetic testing for patients with hematuria is one way to accomplish this objective. In our view, cascade screening of individuals with additional clinical indicators—such as coexisting ocular or auditory abnormalities, a family history of Alport syndrome, or a family history of urinary abnormalities or kidney dysfunction even in the absence of a confirmed diagnosis—may demonstrate greater efficacy and diagnostic yield than universal screening. Our study did not evaluate the effectiveness of urine screening in all children aged 3 years for the identification of Alport syndrome because only those with Alport syndrome were examined in the present cohort. Future investigations are warranted to assess the diagnostic yield and clinical utility of cascade screening for genetically confirmed Alport syndrome. In the future, if genetic screening for identifying disease in infants and young children becomes widespread, it could enable the early detection of causative gene variants in Alport syndrome. However, as of 2024, universal implementation remains challenging because of ethical considerations, costs, and other factors. Urinary screening is considered an optimal economical method for contributing to the early detection of Alport syndrome as an alternative to genetic screening. A previous study suggested that, for Alport syndrome, where early intervention can improve prognosis of the kidney, implementing a screening system capable of facilitating early detection—and thus enabling early intervention—would offer considerable benefits.^13^

Many years have passed since the contribution of RAS-I therapy delaying ESKD in Alport syndrome was demonstrated. Yamamura *et al.*^20^ reported that RAS-I treatment in male adults with XL Alport syndrome delayed the onset of ESKD by > 20 years. Similarly, Zhang *et al.*^30^ found that the administration of RAS-Is to adults with AR Alport syndrome delayed the progression to ESKD by > 10 years. Therefore, evidence has shown that the administration of RAS-I therapy to adult patients with Alport syndrome improves prognosis of the kidney. Recently, a randomized controlled trial in Europe adopted a more aggressive therapeutic approach by administering an RAS-I to nearly all pediatric patients with Alport syndrome aged > 2 years (median age: 6 years) at the onset of hematuria alone.^13^ This trial had a relatively short observation period (mean: 4.1 years) and did not set ESKD as an end point.^13^ However, therapeutic intervention in patients with Alport syndrome before the onset of kidney function decline is expected to have a positive effect on prognosis of the kidney. Our findings are in line with the recent ErkNet/ERA/ESPN clinical practice recommendations for the diagnosis and management of Alport syndrome, which emphasize the benefit of early diagnosis and treatment with RAS blockade.^33^ To estimate the total number of children who underwent age-3 urine screening during the study period in Japan, we considered the corresponding birth cohorts. Children who were aged 18 years in 2015 were born in 1997, whereas those who were aged 3 years in 2024 were born in 2021. The cumulative number of live births in Japan during this interval was approximately 26.3 million.^34^ Given the very low mortality rate among infants and young children in Japan, together with the reported participation rate of 86.7% in age-3 urine screening in a 2008 nationwide survey,^14^ we estimate that approximately 22.8 million children underwent screening during the study period.

This study has several limitations. First, there may have been selection bias because we only included patients diagnosed with Alport syndrome genetically. Second, our study was a retrospective investigation. Third, we were unable to collect clinical data, such as patients’ blood pressure and kidney imaging findings. Fourth, potential disadvantages of universal urine screening include false-positive results, which may lead to unnecessary follow-up visits, additional laboratory testing, increased health care expenditures, and heightened anxiety for both parents and children. These factors should be carefully considered when implementing or revising population-based screening programs. Finally, this study did not verify the kidney prognosis of Alport syndrome diagnosed at the age-3 urine screening. Therefore, we plan to continue monitoring the progression of Alport syndrome in our patients.

### Conclusion

The most common setting for diagnosing Alport syndrome was age-3 urine screening. More than half of the patients genetically diagnosed with Alport syndrome detected through the age-3 urine screening were already eligible for RAS-I therapy at the timing of the screening. Urinary screening might be an optimal method for contributing to the early detection of Alport syndrome.

## Disclosure

KN is a member of advisory groups for Kyowa Kirin Co. Ltd., Toa Eiyo Ltd., Zenyaku Kogyo Co. Ltd., and Taisho Pharmaceutical Co. Ltd; received lecture fee from Ono Pharma, Astellas Pharma, Novo Nordisk Pharma, Alexion Pharma, Sumitomo Pharma, Sanofi, Otsuka Pharma, Daiichi Sankyo, Miyarisan; received a grant from Zenyaku Kogyo and Torii Co. Ltd; and has a patent for developing exon skipping therapy for Alport syndrome. All the other authors declared no competing interests.

## Supplementary Material

Supplementary File (PDF) **Table S1.** The characteristics of patients with Alport syndrome genetically through age-3 urine screening.
