# Supplementary material for: Impact of Age-3 Urine Screening on Diagnosis and Treatment Timing in Alport Syndrome
Source: Kidney Int Rep. 2025 Sep 23;10(12):4234–40. doi: 10.1016/j.ekir.2025.09.022 (PMC12712584; doi:10.1016/j.ekir.2025.09.022)
Supplement: Supplementary File (PDF) — Table S1. The characteristics of patients with Alport syndrome genetically through age-3 urine screening. [file mmc1.pdf]

ID	Sex	Age (y)	Proteinuria (g/gCr)	Cr-eGFR (mL/min/1.73m^2)	Family history	Gene	Exon/ Intron	Mutation	Amino Acid	Genotype (NT/T/S)	Previously reported	ACMG criteria (P/LP, details)	
A341	F	4	0.10	100.8	Alport syndrome	*COL4A5*	Exon 25	c.1897G>A	Glu633Lys	NT	Inoue Y et al [1].	P	PS1, PM1, PM2, PP1, PP3, PP4
A346	M	10	0.04	143.3	-	*COL4A3*	Exon 7	c.424G>A	Gly142Ser	NT	-	LP	PM1, PM2, PP1, PP2, PP3
A348	M	4	0.32	NA	-	*COL4A5*	Exon 23	c.1526G>A	Gly509Asp	NT	Hashimura Y et al [2].	LP	PS1, PM1, PM2, PP3
A365	M	4	1.60	165.5	-	*COL4A5*	Intron 40	c.3604+1G>A	-	S	-	P	PSV1, PM2
A370	M	6	1.50	NA	ESKD	*COL4A5*	Exon 23	c.1547delA	-	T	-	P	PSV1, PM2
A374	F	14	0.17	98.0	-	*COL4A5*	Exon 46	c.4279G>A	Gly1427Ser	NT	Di H et al [3].	LP	PS1, PM1, PM2, PP3
A378	M	12	0.33	138.6	ESKD	*COL4A5*	Exon 21	c.1357_1365del CCAGGCCCT	-	NT	-	LP	PM1, PM2, PM4
A395	F	15	0.31	85.9	-	*COL4A5*	Exon 34	c.2999G>T	Gly1000Val	NT	-	LP	PM1, PM2, PP1, PP3, PP4, BP6
A406	F	4	0.25	135.3	-	*COL4A5*	Exon 11	c.638G>T	Gly213Val	NT	-	LP	PM1, PM2, PM5, PP3
A413	M	17	2.17	12.9	ESKD	*COL4A5*	Exon 13	c.688G>T	Gly230Cys	NT	Kamura M et al [4].	LP	PS1, PM2, PP3, PP4
A415	F	17	0.35	107.7	-	*COL4A3*	Exon 48	c.4441C>T	Arg1481*	T	Lemmink HH et al [5].	LP	PS1, PM2, PP3, PP4
A424	M	10	0.32	76.3	ESKD/Alport syndrome	*COL4A5*	Intron 6	c.385-1G>A	-	S	-	P	PSV1, PS3, PM2
A426	F	3	0.29	108.1	-	*COL4A5*	Exon 13	c.716G>A	Gly239Glu	NT	Knebelmann B et al [6].	LP	PS1, PM2, PP3
A436	F	4	0.66	84.4	-	*COL4A5*	Exon 48	c.4592C>G	Ser1531*	T	-	P	PSV1, PM2
A444	M	7	1.62	147	-	*COL4A4*	Exon 47	c.4768C>T	Gln1590*	T	Groopman EE et al [7].	P	PSV1, PS1, PM2
A448	M	11	0.12	148.83	-	*COL4A5*	Exon 47	c.4325G>C	Gly1442Ala	NT	Höpker K et al [8].	P	PS1, PM1, PM2, PP1, PP3
A452	F	14	0.36	109.5	-	*COL4A5*	Intron 29	c.2395+5G>A	-	S	-	LP	PS3, PM2
A457	F	13	NA	71.4	-	*COL4A3*	Exon 15	c.833dupT	Pro279Alafs*8	T	Zhang Y et al [9].	P	PVS1, PS1, PM2, PP5
A458	F	5	0.41	127.0	-	*COL4A5*		Deletion of Exon 2-32			-	P	PVS1, PS3, PP4
A477	F	6	0.26	NA	-	*COL4A3*	Exon 18	c.991C>T	Gln331*	T	-	P	PVS1, PM2, PP5
A478	F	6	0.08	136.0	ESKD	*COL4A5*	Exon 29	c.2332G>A	Gly778Ser	NT	Jayasinghe K et al [10].	P	PS1, PM1, PM2, PP1, PP3
A484	F	13	1.81	194.8	-	*COL4A5*	Exon 17	c.946G>T	Gly316Cys	NT	-	LP	PM1, PM2, PM5, PP3
A487	M	4	0.32	149.9	ESKD	*COL4A5*	Exon 20	c.1189G>A	Gly397Ser	NT	-	LP	PM1, PM2, PP3, PP4
A489	F	4	0.85	178.2	-	*COL4A5*	Exon 42	c.3905delA	Gly1303Aspfs*17	T	-	P	PVS1, PM2
A491	F	4	0.16	143.0	-	*COL4A5*		Deletion of Exon 16-18, 50-51			-	P	PVS1, PM2
A493	M	8	0.77	135.4	-	*COL4A5*	Exon 11	c.638G>A	Gly213Glu	NT	King K et al [11].	P	PS1, PM1, PM2, PM5, PP3
A506	F	6	0.41	164.5	ESKD/Alport syndrome	*COL4A5*	Exon 47	c.4353delT	Pro1453Leufs*95	T	-	P	PVS1, PM2
A511	M	4	10.5	123.1	-	*COL4A5*	Exon 50	c.4819dupG	Ala1607Glyfs*33	T	-	P	PVS1, PM2
A515	F	7	0.12	135.0	ESKD	*COL4A5*	Exon 41	c.3685G>A	Gly1229Ser	NT	Hanson H et al [12].	P	PS1, PM1, PM2, PP1, PP3, PP4
A516	F	3	0.95	152.51	ESKD	*COL4A5*	Exon 50	c.4974C>T	-	S	-	LP	PS3, PM2, PP4, BP4
A526	F	6	0.32	146.9	-	*COL4A5*	Exon 45	c.4157_4158 insTA	Gly1388Argfs*16 1	T	-	P	PVS1, PM2
A527	M	7	0.30	NA	-	*COL4A5*	Exon 1	c.41_44 delTACT	Leu14Trpfs*3	T	-	P	PVS1, PM2, PM4
A538	M	13	8.90	168.8	-	*COL4A4*	Exon 25	c.1862dupC	Gly622Argfs*35	T	-	P	PSV1, PM2
A571	F	14	0.06	122.7	ESKD	*COL4A5*	Exon 29	c.2332G>A	Gly778Ser	NT	Plant KE et al [13].	P	PS1, PM1, PM2, PP1, PP3
A573	F	12	0.26	85.0	-	*COL4A5*	Exon 9	c.494C>G	Ser165*	T	-	P	PVS1, PM2, PP5
A591	F	18	0.53	115.7	-	*COL4A4*	Intron 47	c.4809+5G>A		S	-	LP	PS3, PM2, PP1
A592	M	4	0.30	165.2	-	*COL4A5*	Exon 8	c.458G>A	Gly153Asp	NT	Bekheirnia MR et al [14].	LP	PS1, PM1, PM2, PP3
A599	F	11	0.11	111.2	-	*COL4A5*	Exon 41	c.3703G>A	Gly1235Ser	NT	-	LP	PM1, PM2, PP3, PP4
A609	M	5	0.07	124.0	Alport syndrome	*COL4A5*	Exon 28	c.2215C>G	Pro73Ala	NT	Fang w et al.	US	PS1, PM1, BP4
A612	F	9	0.07	97.7	ESKD/Alport syndrome	*COL4A5*	Exon 35	c.3035G>A	Gly1012Asp	NT	Di H et al [3].	P	PS1, PM1, PM2, PP3, PP4
A615	M	3	0.15	140.7	-	*COL4A5*	Intron 18	c.1032+5G>C		S	-	LP	PS3, PM2
A651	M	5	0.26	122.3	ESKD	*COL4A5*	Exon 40	c.3596G>A	Gly1199Glu	NT	Seo GH et al [15].	LP	PS1, PM1, PM2, PP3
A653	M	13	0.40	121.4	-	*COL4A3*	Exon 33	c.2696C>T	Ala899Val	NT	-	US	PM1, PM2, BP4
A664	F	8	0.30	112.6	ESKD	*COL4A5*	Exon 42	c.3809G>T	Gly1270Val	NT	-	LP	PM1, PM2, PM5, PP3
A686	M	5	0.26	118.0	ESKD/Alport syndrome	*COL4A5*	Exon 16	c.893G>A	Gly298Asp	NT	Hashimura Y et al [2].	LP	PS1, PM1, PM2, PP3
A694	M	15	0.08	120.3	ESKD	*COL4A5*	Exon 34	c.2999G>T	Gly1000Val	NT	-	LP	PM1, PM2, PP1, PP3, PP4, BP6

A698	F	10	0	110.6	-	*COL4A5*	Exon 25	c.1825G>A	Gly609Ser	NT	-	LP	PM1, PM2, PM5, PP3
A713	F	15	0.38	148.8	-	*COL4A5*	Intron 12	c.687+5G>C	-	S	-	LP	PS3, PM2
A715	F	7	2.14	138.1	-	*COL4A5*	Exon 36	c.3143G>T	Gly1048Val	NT	-	LP	PS2, PM1, PM2, PP3
A722	M	11	2.10	99.9	ESKD	*COL4A5*	Exon 10	c.548G>A	Gly183Asp	NT	-	LP	PM1, PM2, PM5, PP3
A725	F	4	0.20	100.0	-	*COL4A5*	Exon 15	c.874G>C	Gly292Arg	NT	Barker DF et al [16].	P	PS1, PM1, PM2, PP1, PP3, PP4
A733	F	4	0.13	150.5	-	*COL4A5*	Exon 28	c.2213delC	Pro738Leufs*54	T	-	P	PVS1, PM2
A736	F	5	0.18	120.0	-	*COL4A5*	Exon 50	c.4808_4809 insGT	Ser1604*	T	-	P	PVS1, PM2
A741	F	7	8.14	132.3	-	*COL4A4*	Exon 24	c.1795G>C	Gly599Arg	NT	-	LP	PM1, PM2, PM5, PP1, PP3, BP4
A747	F	4	0.27	131.4	ESKD	*COL4A3*	Exon 23	c.1496G>A	Gly499Glu	NT	Zhang Y et al [9].	LP	PS1, PM1, PM2, PP3
A782	M	17	1.79	82.4	-	*COL4A5*	Exon 27	c.2105G>A	Gly702Asp	NT	Di H et al [3].	P	PS1, PM1, PM2, PP3, PP4
A792	F	3	0.77	155.1	Alport syndrome	*COL4A5*	Exon 51	c.5029C>T	Arg1677*	T	Plant KE et al [13].	P	PSV1, PS1, PM2, PP1, PP4
A797	F	6	0.05	139.8	-	*COL4A5*	Exon 38	c.3427G>A	Gly1143Ser	NT	Renieri A et al [17].	P	PS1, PM1, PM2, PP1, PP3, PP4
A800	M	4	0.26	133.5	-	*COL4A5*	Intron 2	c.143-2A>G	-	S	-	P	PVS1, PM2
A842	M	3	1.91	131.8	-	*COL4A3*	Exon 1	c.40_63delCTGC CGCTCCTGCTG GTGCTCCTG	Leu14_Leu21del	NT	Longo I et al [18].	LP	PS1, PM2, PM4, BP3
A854	F	10	0.13	130.0	ESKD	*COL4A5*		Deletion of Exon 35-36			-	P	PVS1, PM2
A858	M	14	0.19	107.5	ESKD	*COL4A5*	Exon 35	c.3035G>A	Gly1012Asp	NT	Di H et al [3].	P	PS1, PM1, PM2, PP3, PP4
A864	F	5	1.49	153.0	-	*COL4A5*	Exon 29	c.2394A>T	Leu798Asn	NT	-	LP	PS2, PM1, PM2, PP3
A865	F	16	0.58	137.7	-	*COL4A5*	Intron 46	c.4298-14_4298-9del	-	S	-	US	PM2, PP3, PP4
A866	F	4	0.21	119.0	-	*COL4A5*	Intron 9	c.546+2_546+3 insTT	-	S	Hashimura Y et al [2].	P	PVS1, PS1, PM2
A870	M	14	5.87	100.6	-	*COL4A5*	Intron 29	c.2396-1G>A	-	S	-	P	PVS1, PM2, PP5
A882	M	5	0.21	134.9	-	*COL4A3*	Exon 21	c.1229G>A	Gly410Glu	NT	-	LP	PM1, PM2, PM3, PP3
						COL4A3	Intron 18	c.1030-2A>C	-	S	-	P	PVS1, PM2
A887	M	4	0.30	100.0	-	*COL4A5*	Exon 20	c.1217G>A	Gly406Asp	NT	Hanson H et al [12].	P	PS1, PM1, PM2, PM5, PP3
A907	M	15	0.09	99.7	-	*COL4A5*	Exon 19	c.1075G>C	Gly359Arg	NT	Yamamura T et al [19].	LP	PS1, PM2, PP1, PP3
A914	F	4	0.31	90.5	-	*COL4A5*	Exon 42	c.3920_3923 delinsACT	Leu1307Hisfs*13	T	-	P	PVS1, PM2
A916	F	8	0.11	111.7	-	*COL4A4*	Exon 33	c.3086delC	Pro1029Glnfs*9	T	-	P	PVS1, PM2
A919	F	14	0.18	101.1	Alport syndrome	*COL4A5*	Exon 35	c.3035G>A	Gly1012Arg	NT	Di H et al [3].	P	PS1, PM1, PM2, PP3, PP4
A941	F	4	0.18	115.8	ESKD	*COL4A5*	Exon 31	c.2554G>A	Gly852Arg	NT	Kawai S et al [20].	P	PS1, PM1, PM2, PP3, PP4
A960	M	4	1.29	98.3	ESKD	*COL4A5*	Exon 25	c.1808G>C	Gly603Ala	NT	-	LP	PM1, PM2, PM5, PP3
A994	M	14	NA	NA	-	*COL4A4*	Exon 44	c.4129C>T	Arg1377*	T	Boye E et al [21].	P	PVS1, PS1, PM2, PP1
A1000	M	4	0.39	NA	-	*COL4A5*	Exon 2	c.114_115delTG	Cys38*	T	-	P	PVS1, PM2
A1001	F	3	0.58	129.6	-	*COL4A5*	Exon 31	c.2605G>A	Gly869Arg	NT	Boye E et al [22].	P	PS1, PM1, PM2, PP1, PP3
A1007	M	3	0.64	114.0	Alport syndrome	*COL4A5*	Exon 33	c.2902dupG	Glu968Glyfs*43	T	Yamamura T et al [19].	P	PVS1, PS1, PM2
A1008	F	4	0.09	109.0	-	*COL4A5*	Exon 23	c.1526G>A	Gly509Asp	NT	Hashimura Y et al [2].	LP	PS1, PM1, PM2, PP3
A1017	M	4	2.07	106.1	-	*COL4A4*	Exon 22	c.1476_1477 delTG	Ala493Leufs*2	T	-	P	PVS1, PM2
						COL4A4	Exon 30	c.2608G>C	Gly870Arg	NT	Oka M et al [23].	P	PS1, PM1, PM2, PM3, PP3
A1030	M	11	0.16	107.0	-	*COL4A3*	Exon 37	c.3079G>C	Gly1027Arg	NT	-	LP	PM1, PM2, PP2, PP3
A1035	M	14	0.13	105.2	ESKD	*COL4A4*	Exon 29	c.2384G>A	Gly795Gln	NT	-	LP	PM2, PM5, PP1, PP3
A1044	F	4	0.08	112.1	ESKD	*COL4A3*	Exon 28	c.2125G>T	Gly709*	T	Oka M et al [23].	P	PVS1, PM2
A1050	F	4	0.30	96.1	-	*COL4A5*	Intron 34	3017-1G>A	-	S	Barker DF et al [16].	P	PVS1, PS1, PM2
A1051	F	4	0.10	116.3	ESKD/Alport syndrome	*COL4A5*	Exon 29	2332G>A	Gly778Ser	NT	Plant KE et al [13].	P	PS1, PM1, PM2, PP1, PP3, PP4
A1064	F	3	0.94	141.0	Alport syndrome	*COL4A5*	Exon 20	1225G>A	Gly409Ser	NT	Sun L et al [24].	P	PS1, PM1, PM2, PP1, PP3, PP4
A1072	F	4	0.11	112.4	ESKD	*COL4A3*	Exon 42	3592G>C	Gly1198Arg	NT	Longo I et al [18].	P	PS1, PM1, PM2, PM5, PP3
A1084	M	14	2.53	66.7	-	*COL4A5*	Exon 47	4350delA	Gly1451Valfs*97	T	-	P	PVS1, PM2

A1097	F	4	NA	NA	Alport syndrome	*COL4A4*	Exon 25	1807dupG	Asp603Glyfs*3	T	-	P	PVS1, PM2
A1114	F	18	0.41	163.8	-	*COL4A5*	Intron 1	c.81+2dupT	-	S	-	P	PVS1, PM2
A1115	M	11	0.06	92.5	ESKD	*COL4A3*	Exon 32	c.2620G>C	Gly874Arg	NT	-	LP	PM1, PM2, PP2, PP3
A1124	M	7	0.12	165.2	ESKD	*COL4A5*	Exon 19	1075G>C	Gly359Arg	NT	Yamamura T et al [19].	LP	PS1, PM2, PP1, PP3
						COL4A4	Exon 14	c.827G>C	Gly276Ala	NT	Imafuku A et al [25].	LP	PS1, PM2, PP3
A1143	F	15	0.34	118.5	ESKD	*COL4A3*	Intron 24	c.1576-20_1576-6delTCATTTGTGTACTAC	-	S	Oka M et al [23].	P	PS1, PS3, PM2, PM4
A1144	M	5	0.17	135.4	-	*COL4A4*	Exon 44	c.4195A>T	Met1399Leu	T	Liwei G et al.	US	PS1, PM2, PP1, BP4, BP6
A1171	F	16	0.14	103.0	-	*COL4A4*	Exon 19	c.1108G>C	Gly370Arg	NT	-	LP	PM1, PM2, PM5, PP3
A1173	F	5	0.26	111.2	-	*COL4A3*	Exon 19	c.1087G>A	Gly363Arg	NT	-	LP	PM1, PM2, PP3, PP5
A1198	M	6	0.11	121.4	ESKD	*COL4A3*	Exon 25	c.1751G>A	Gly584Asp	NT	-	LP	PM1, PM2, PM5, PP3
A1202	F	4	0.19	109.0	ESKD	*COL4A4*	Intron 2	c.71+1G>A	-	S	Nabais Sá MJ et al [26].	P	PVS1, PS1, PM2, PP5
						COL4A4	Exon 27	c.2084G>A	Gly695Asp	NT	Oka M et al [23].	P	PS1, PM1, PM2, PP1, PP3
A1211	F	9	0.18	233.3	-	*COL4A3*	Intron 35	c.2981-2A>C	-	S	-	P	PVS1, PM2
A1233	M	4	0.39	124.8	-	*COL4A5*	Exon 48	c.4688G>A	Arg1563Gln	NT	Zhou J et al [27].	P	PS1, PM1, PM2, PP1, PP3
A1294	M	9	0	134.2	ESKD	*COL4A3*	Exon27	c.1976G>A	Gly659Glu	NT	-	US	PM2, PP3
A1266	F	5	0.27	128.4	-	*COL4A5*	Exon 41	c.3612delG	Gly1025Valfs*94	T	-	P	PVS1, PM2
A1273	F	9	0.18	106.9	ESKD	*COL4A5*	Intron 6	c.385-1G>A	-	S	-	P	PVS1, PS3, PM2
A1276	F	14	0.08	91.5	ESKD	*COL4A5*	Exon 17	c.973G>A	Gly325Arg	NT	Knebelmann B et al [28].	P	PS1, PM1, PM2, PP1, PP3, PP4
A1286	F	10	0.73	115.0	-	*COL4A5*	Exon 26	c.1987G>C	Gly663Arg	NT	-	LP	PM1, PM2, PP3, PP4
A1296	M	13	0.12	113.0	-	*COL4A5*	Exon 10	c.601G>C	Gly201Arg	NT	Sun L et al [29].	LP	PS1, PM1, PM2, PP3
A1313	M	5	0.04	133.5	ESKD	*COL4A5*	Exon 1	c.44T>C	Leu15Pro	NT	-	US	PM2, PP1, PP3, PP4
A1327	F	4	0.35	139.0	-	*COL4A5*	Exon 30	c.2503C>T	Gln835*	T	-	P	PVS1, PM2
A1328	F	5	0.17	142.0	-	*COL4A5*	Exon 28	c.2236G>T	Gly746*	T	-	P	PVS1, PM2
A1337	M	4	0.10	124.0	-	*COL4A5*	Exon 19	c.1075G>C	Gly359Arg	NT	Yamamura T et al [19].	LP	PS1, PM2, PP1, PP3
A1345	M	9	0.13	119.3	-	*COL4A5*	Exon 47	c.4352G>A	Gly1451Asp	NT	Morinière V et al [30].	LP	PS1, PM1, PM2, PP3
A1363	M	9	0.14	95.0	-	*COL4A3*		Deletion of Exon 11-18			-	P	
A1377	F	3	0.99	147.8	-	*COL4A5*	Intron 46	c.4298-2A>T	-	S	-	P	PVS1, PM2

Supplemental Table 1: The characteristics of patients with Alport syndrome genetically through age-3 urine screening

Cr-eGFR: Creatinine-estimated glomerular filtration rate, M: Male, F: Female, NA: Not applicable, ESKD: End-stage kidney disease, T: Truncating, NT: Non-truncating, S: Splicing,

P: Pathogenic, LP: Likely pathogenic, US: Uncertain significance

Supplementary References

[S1] Inoue Y, Nishio H, Shirakawa T, Nakanishi K, Nakamura H, Sumino K, et al. Detection of mutations in the COL4A5 gene in over 90% of male patients with X-linked Alport's syndrome by RT-PCR and direct sequencing. Am J Kidney Dis. 1999;34:854-62.

[S2] Hashimura Y, Nozu K, Kaito H, Nakanishi K, Fu XJ, Ohtsubo H, et al. Milder clinical aspects of X-linked Alport syndrome in men positive for the collagen IV α5 chain. Kidney Int. 2014;85:1208-13.

[S3] Di H, Zhang J, Gao E, Zheng C, Huang X, Wang Q, et al. Dissecting the genotype-phenotype correlation of COL4A5 gene mutation and its response to renin-angiotensin-

aldosterone system blockers in Chinese male patients with Alport syndrome. Nephrol Dial Transplant. 2022;37:2487-95.
[S4] Kamura M, Yamamura T, Omachi K, Suico MA, Nozu K, Kaseda S, et al. Trimerization and Genotype-Phenotype Correlation of COL4A5 Mutants in Alport Syndrome. Kidney Int Rep. 2020;5:718-26.
[S5] Lemmink HH, Mochizuki T, van den Heuvel LP, Schröder CH, Barrientos A, Monnens LA, et al. Mutations in the type IV collagen alpha 3 (COL4A3) gene in autosomal recessive Alport syndrome. Hum Mol Genet. 1994;3:1269-73.
[S6] Knebelmann B, Breillat C, Forestier L, Arrondel C, Jacassier D, Giatras I, et al. Spectrum of mutations in the COL4A5 collagen gene in X-linked Alport syndrome. Am J Hum Genet. 1996;59:1221-32.
[S7] Groopman EE, Marasa M, Cameron-Christie S, Petrovski S, Aggarwal VS, Milo-Rasouly H, et al. Diagnostic Utility of Exome Sequencing for Kidney Disease. N Engl J Med. 2019;380:142-51.
[S8] Höpker K, Liebau MC, Friederichsohn C, Waldherr R, Benzing T. Atypical Alport syndrome associated with a novel COL4A5 mutation. Clin Nephrol. 2009;71:321-5.
[S9] Zhang Y, Böckhaus J, Wang F, Wang S, Rubel D, Gross O, et al. Genotype-phenotype correlations and nephroprotective effects of RAAS inhibition in patients with autosomal recessive Alport syndrome. Pediatr Nephrol. 2021;36:2719-30.
[S10] Jayasinghe K, Stark Z, Kerr PG, Gaff C, Martyn M, Whitlam J, et al. Clinical impact of genomic testing in patients with suspected monogenic kidney disease. Genet Med. 2021;23:183-91.
[S11] King K, Flinter FA, Green PM. A two-tier approach to mutation detection in the COL4A5 gene for Alport syndrome. Hum Mutat. 2006;27:1061.
[S12] Hanson H, Storey H, Pagan J, Flinter F. The value of clinical criteria in identifying patients with X-linked Alport syndrome. Clin J Am Soc Nephrol. 2011;6:198-203.
[S13] Plant KE, Green PM, Vetrie D, Flinter FA. Detection of mutations in COL4A5 in patients with Alport syndrome. Hum Mutat. 1999;13:124-32.
[S14] Bekheirnia MR, Reed B, Gregory MC, McFann K, Shamshirsaz AA, Masoumi A, et al. Genotype-phenotype correlation in X-linked Alport syndrome. J Am Soc Nephrol. 2010;21:876-83.
[S15] Seo GH, Kim T, Choi IH, Park JY, Lee J, Kim S, et al. Diagnostic yield and clinical utility of whole exome sequencing using an automated variant prioritization system, EVIDENCE. Clin Genet. 2020;98:562-70.
[S16] Barker DF, Denison JC, Atkin CL, Gregory MC. Efficient detection of Alport syndrome COL4A5 mutations with multiplex genomic PCR-SSCP. Am J Med Genet. 2001;98:148-60.
[S17] Renieri A, Meroni M, Sessa A, Battini G, Serbelloni P, Torri Tarelli L, et al. Variability of clinical phenotype in a large Alport family with Gly 1143 Ser change of collagen alpha 5(IV)-chain. Nephron. 1994;67:444-9.
[S18] Longo I, Porcedda P, Mari F, Giachino D, Meloni I, Deplano C, et al. COL4A3/COL4A4 mutations: from familial hematuria to autosomal-dominant or recessive Alport syndrome. Kidney Int. 2002;61:1947-56.
[S19] Yamamura T, Nozu K, Fu XJ, Nozu Y, Ye MJ, Shono A, et al. Natural History and Genotype-Phenotype Correlation in Female X-Linked Alport Syndrome. Kidney Int Rep. 2017;2:850-5.

[S20] Kawai S, Nomura S, Harano T, Harano K, Fukushima T, Osawa G. The COL4A5 gene in Japanese Alport syndrome patients: spectrum of mutations of all exons. The Japanese Alport Network. Kidney Int. 1996;49:814-22.

[S21] Boye E, Mollet G, Forestier L, Cohen-Solal L, Heidet L, Cochat P, et al. Determination of the genomic structure of the COL4A4 gene and of novel mutations causing autosomal recessive Alport syndrome. Am J Hum Genet. 1998;63:1329-40.

[S22] Boye E, Flinter F, Zhou J, Tryggvason K, Bobrow M, Harris A. Detection of 12 novel mutations in the collagenous domain of the COL4A5 gene in Alport syndrome patients. Hum Mutat. 1995;5:197-204.

[S23] Oka M, Nozu K, Kaito H, Fu XJ, Nakanishi K, Hashimura Y, et al. Natural history of genetically proven autosomal recessive Alport syndrome. Pediatr Nephrol. 2014;29:1535-44.

[S24] Sun L, Zhang J, Kuang XY, Kang YL, Wu Y, Huang WY. Generation of an induced pluripotent stem cell line (SHCDNRi001-A) from a patient with X-linked Alport syndrome carrying a heterozygous p.G409S (c. 1225 G > A) mutation in the COL4A5 gene. Stem Cell Res. 2020;45:101833.

[S25] Imafuku A, Nozu K, Sawa N, Hasegawa E, Hiramatsu R, Kawada M, et al. Autosomal dominant form of type IV collagen nephropathy exists among patients with hereditary nephritis difficult to diagnose clinicopathologically. Nephrology (Carlton). 2018;23:940-7.

[S26] Nabais Sá MJ, Storey H, Flinter F, Nagel M, Sampaio S, Castro R, et al. Collagen type IV-related nephropathies in Portugal: pathogenic COL4A3 and COL4A4 mutations and clinical characterization of 25 families. Clin Genet. 2015;88:456-61.

[S27] Zhou J, Gregory MC, Hertz JM, Barker DF, Atkin C, Spencer ES, et al. Mutations in the codon for a conserved arginine-1563 in the COL4A5 collagen gene in Alport syndrome. Kidney Int. 1993;43:722-9.

[S28] Knebelmann B, Deschenes G, Gros F, Hors MC, Grünfeld JP, Zhou J, et al. Substitution of arginine for glycine 325 in the collagen alpha 5 (IV) chain associated with X-linked Alport syndrome: characterization of the mutation by direct sequencing of PCR-amplified lymphoblast cDNA fragments. Am J Hum Genet. 1992;51:135-42.

[S29] Sun L, Kuang X, Hao S, Wang P, Niu X, Zhu G, et al. [Features of clinical phenotype and genotype in Alport syndrome: a monocentric study]. Zhonghua Er Ke Za Zhi. 2015;53:114-8.

[S30] Morinière V, Dahan K, Hilbert P, Lison M, Lebbah S, Topa A, et al. Improving mutation screening in familial hematuric nephropathies through next generation sequencing. J Am Soc Nephrol. 2014;25:2740-51.
